# Circular RNA hsa_circ_0001846 facilitates the malignant behaviors of pancreatic cancer by sponging miR-204-3p and upregulating KRAS expression

**DOI:** 10.1038/s41420-023-01733-2

**Published:** 2023-12-11

**Authors:** Xiaolei Ma, Haocheng Zhang, Luning Wang, Mengxing Cheng, Yanxin Jia, Di Feng, Yating Lei, Xinyu Li, Lina Ba, Na Song, Xinxia Yang, Lidan Bai, Ruipu Zhang, Wenxiao Xu, Guofen Qiao

**Affiliations:** 1 Department of Pharmacology (State-Province Key Laboratories of Biomedicine-Pharmaceutics of China, Key Laboratory of Cardiovascular Research, Ministry of Education), College of Pharmacy, Harbin Medical University, Harbin, 150086 China; 2 Department of Pharmacy, the Sixth Affiliated Hospital of Harbin Medical University, Harbin, 150086 China; 3 Department of pharmacy intravenous admixture services, the Second Affiliated Hospital of Harbin Medical University, Harbin, 150086 China; 4 Department of Pathology, Harbin Medical University Cancer Hospital, Harbin, 150086 China; 5 Department of Orthopedics, the Second Affiliated Hospital of Harbin Medical University, Harbin, 150086 China

**Keywords:** Targeted therapies, Long non-coding RNAs

## Abstract

Pancreatic cancer (PC) is mainly derived from the exocrine pancreatic ductal epithelial cells, and it is strongly aggressive malignant tumor. Due to its insidious onset and the lack of effective diagnostic biomarkers, PC currently remains one of the main causes of cancer-related mortality worldwide. Recent studies have found that hsa_circ_0001846 is involved in the progression of multiple cancers and has the potential to become biomarkers, but its function and mechanism in PC remains unclear. We found by qRT-PCR experiments that hsa_circ_0001846 was upregulated in PC cells and tissues, while circBase, Sanger sequencing, agarose gel electrophoresis and FISH experiments identified the splicing site, ring structure and cellular localization of hsa_circ_0001846. Various functional experiments by using the construction of small interfering RNA targeting hsa_circ_0001846 and overexpression plasmid demonstrated that hsa_circ_0001846 promoted the proliferation, migration and invasion of PC cells. Moreover, the tumor weight and volume of nude mice were significantly reduced after the stable knockdown of hsa_circ_0001846. In the mechanism exploration, RNA pull-down experiments and dual-luciferase experiments helped us to determine that hsa_circ_0001846 regulated the KRAS expression by sponging miR-204-3p in PC, thus playing a pro-cancer role. In this study, the effect of miR-204-3p on PC was also explored for the first time, and we found that knockdown of miR-204-3p reversed the tumor suppressive effect caused by silencing hsa_circ_0001846, and silencing KRAS also rescued the pro-cancer effect caused by overexpression of hsa_circ_0001846. In conclusion, our study revealed the pro-cancer role of hsa_circ_0001846 in PC, and for the first time identified the mechanism that hsa_circ_0001846 regulated KRAS by sponging miR-204-3p to promote PC progression and had the potential to become a cancer biomarker.

## Introduction

Pancreatic cancer (PC) can be caused by endocrine or exocrine cells, is by far the most lethal pancreatic disease and its etiology is usually associated with other pancreatic diseases [1]. Although its 5-year survival rate has increased to 10% in the past few years, PC remains one of the deadliest types of human cancer [2]. PC kills 200,000 people worldwide each year, and the incidence of PC is higher in developed countries than in developing countries [3]. At present, surgery is the only means to cure PC. However, due to the characteristics of hidden onset and rapid invasion of PC, 80% of patients have entered the middle and late stage when they seek medical treatment, only 15–20% of patients have indications for surgery, and patients possibly still need to face the risk of recurrence. For patients with local metastasis and systemic metastasis, only systemic chemotherapy can be relied on to prolong life. The early clinical chemotherapy regimen is gemcitabine (GEM) alone treatment [4]. In recent years, FOLFIRINOX combination chemotherapy regimen with multiple drugs has shown better efficacy [4]. However, chemotherapy is often less effective because PC has acquired strong cellular protective mechanisms that increase drug resistance [5]. All these factors make the early diagnosis of PC particularly important, which is a major problem to be solved urgently in clinic. Although there have been some diagnostic and therapeutic achievements in the fight against PC, we still need to further explore the pathogenesis of PC and potential therapeutic targets.

Circular RNA (CircRNA) is a class of non-coding RNA with a closed ring structure, without 5’cap structure and 3’poly A tail, which makes them more resistant to digestion of RNase R and more stable than linear RNA. At the same time, they can resist the toxicity of actinomycin D. CircRNAs are generated primarily by reverse shearing, which can be mediated by complementary base pairing of reverse repeating elements (such as Alu elements) located in upstream and downstream introns, or by binding of RBPs or dimers to specific gene sequences of flanking introns [6–10]. There is a competitive relationship between reverse shearing and linear splicing [11]. According to different reverse shearing methods, circRNAs are mainly divided into exon circRNAs, exon-intron circRNAs, intron circRNAs and intergene circRNAs. Exon circRNAs are mainly located in the cytoplasm, exon-intron circRNAs and intron circRNAs are mainly located in the nucleus. During the study, we found that compared with linear RNAs, although the expression level of circRNAs was lower, they still had higher abundance, diversity and specificity, and some circRNAs even had opposite expression level with their homologous linear RNAs in the same tissue or cell [12, 13]. This suggests that circRNAs may have a function of their own.

Current research has found that circRNAs mainly act as regulatory molecules in cells. Existing studies have shown that different expression circRNAs (DECs) can act as oncogenes or tumor suppressors to regulate the proliferation, migration, invasion, metastasis, apoptosis and cell cycle of PC. These functions are related to a variety of mechanisms, including miRNAs sponge activity, regulation of cancer-related signaling pathways, and protein interactions [14]. The upregulated circRNAs of ciRS-7, circFOXK2, hsa_circ_0007534, hsa_circ_100782, circBFAR and circASH2L in PC can sponge miRNAs regulate the expression of downstream related cancer genes to enhance the proliferation and invasion of PC [15–20]. More significantly, some DECs can sponge multiple downstream miRNAs simultaneously, such as circRHOT1, which can simultaneously act as a sponge for miR-26b, miR-125a, miR-330, and miR-382, affecting various cancer-related pathways in PC [21]. CircRNAs plays an important regulatory role in the occurrence and development of PC, and is likely to be used as a biomarker for early diagnosis, filling the gap in the lack of reliable and effective early diagnosis of PC, which is of great significance for improving the overall survival rate. PC is a molecular heterogeneous disease, and the oncogenic gene *KRAS* is a powerful driver of tumor initiation and maintenance. *KRAS* is usually mutated in PC, and the inactivation mutations of tumor suppressor genes such as *CDKN2A/p16*, *TP53,* and *SMAD4* act synergistically with *KRAS* mutations to promote the occurrence and development of PC [22]. Activating KRAS and mutating it were found in about 92% of pancreatic ductal adenocarcinoma (PDAC) and it was a decisive genetic trait for PDAC progression [23]. Although the changes of core genes in the pathogenesis of PC have been well documented, their contribution to the pathogenesis of PC remains to be further explored at the molecular level.

In this study, we found that the hsa_circ_0001846 derived from ubiquitin-associated protein 2 (UBAP2) was highly expressed in PC tissues and cell lines, meanwhile a variety of biological function experiments showed that hsa_circ_0001846 knockdown inhibited the proliferation, invasion and migration abilities of PC to a certain extent, and slowed down the growth of tumor in vivo. Hsa_circ_0001846 affected the binding of miR-204-3p to KRAS by sponging miR-204-3p, while upregulated the expression level of KRAS in the cytoplasm. Meanwhile, KRAS inhibitor canceled the carcinogenic effect of hsa_circ_0001846. These results indicate that hsa_circ_0001846 can promote the progression of PC through miR-204-3p/KRAS pathway, suggesting that hsa_circ_0001846 may be a potential biomarker for clinical diagnosis and treatment of PC.

## Results

### Characterization and expression patterns of hsa_circ_0001846 in PC

**Fig. 1:**
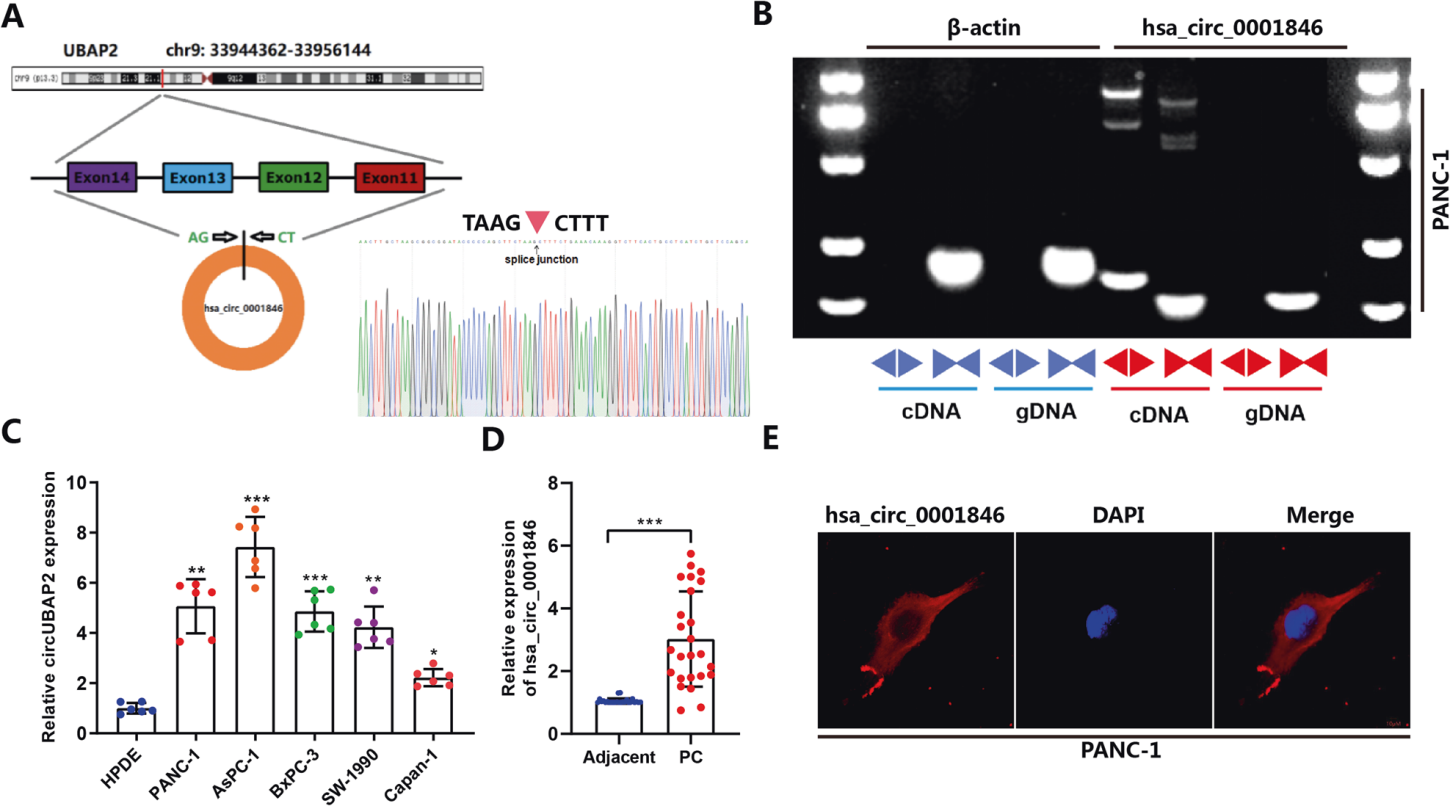
Characterization and expression patterns of hsa_circ_0001846 in PC. **A** The schematic illustration of the gene origin of hsa_circ_0001846 was made according to circBase, and the reverse slicing site of hsa_circ_0001846 was verified by Sanger sequencing. **B** β–actin was used as a control group, divergent and convergent primers were constructed for agarose gel electrophoresis experiments to verify the reverse slicing site and ring structure of hsa_circ_0001846. **C** qRT-PCR experiments were used to analyze the differential expression of hsa_circ_0001846 in five PC cell lines and HPDE cells. **D** The expression level of hsa_circ_0001846 was analyzed by qRT-PCR in 25 pairs of PC tissues. **E** The location of hsa_circ_0001846 in the PC cells was identified by FISH experiment at a Confocal Laser Scanning Microscope. All data are shown as the mean ± SD of at least three independent experiments. **p < 0.01, ***p < 0.001.

To differentiate it from the linear RNA, excluding its effects on the experiments, we have analyzed and verified the structure and properties of hsa_circ_0001846 by querying the circBase software, found that hsa_circ_0001846 originated from the exons 11, 12, 13, and 14 of ubiquitin-associated protein 2 (UBAP2) gene (chr 9:33944362-33956144). The mature sequence was 747 bp and its reverse shear site (TAAGCTTT) was identified by Sanger sequencing (Fig. 1A). Next, we designed qRT-PCR primers and convergent and divergent primers based on the gene sequence of hsa_circ_0001846. The agarose gel electrophoresis experiment showed that successful amplification of hsa_circ_0001846 was only possible by using divergent primers in the cDNA libraries (Fig. 1B). The qRT-PCR results showed that hsa_circ_0001846 was significantly upregulated in various PC cell lines compared to HPDE cells (Fig. 1C). At the same time, we collected 25 pairs of PC tissues, and qRT-PCR results showed that hsa_circ_0001846 had higher expression in cancer tissues than the matched adjacent tissues (Fig. 1D). Moreover, the FISH results indicated that hsa_circ_0001846 was mainly distributed in the cytoplasm of PC cells (Fig. 1E). The above results demonstrated that the UBAP2 gene-derived hsa_circ_0001846 was significantly enriched and stably presented in PC tissues and cytoplasm.

### Hsa_circ_0001846 promotes tumor invasion, proliferation and migration in PC

**Fig. 2:**
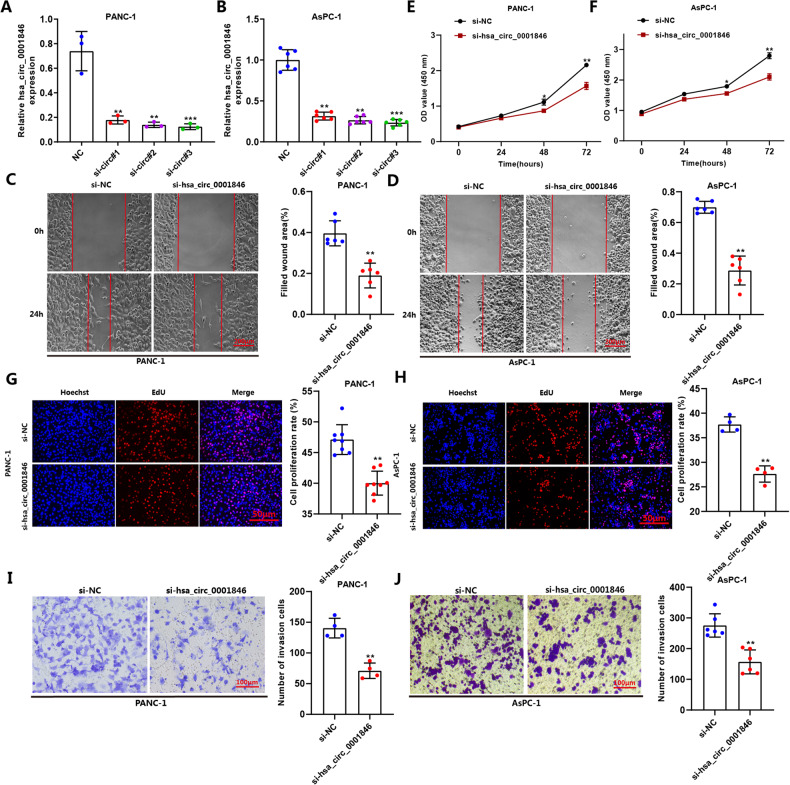
Silencing hsa_circ_0001846 can inhibite biological function of PC cell lines. **A**, **B** Detecting the silencing efficiency of siRNAs in AsPC-1 and PANC-1 by qRT-PCR. **C**–**F** Transfecting si-hsa_circ_0001846 and si-NC in PANC-1 and AsPC-1 to generate wound healing and CCK-8 experiments to explore the cell migration and proliferation abilities. **G**–**J** Transfected PANC-1 or AsPC-1 cells by si-hsa_circ_0001846 or si-NC were used to conduct EdU and Transwell experiments to explore the proliferation and invasion abilities. All data are shown as the mean ± SD of at least three independent experiments. *p < 0.05, **p < 0.01, ***p < 0.001.

Combining the above results, we suspected that hsa_circ_0001846 may play a key role in the malignant progression of PC. Therefore, we investigated the effects in vitro and vivo of hsa_circ_0001846 on migration, proliferation and invasion of PC cells. Firstly, we designed and synthesized three small interfering RNAs (siRNAs) targeting the reverse shear site of hsa_circ_0001846, and verified their silencing efficiency in PANC-1 and AsPC-1 cells. The qRT-PCR results indicated that three siRNAs partially silenced hsa_circ_0001846, but siRNA-3 was most efficiently silenced in PANC-1 and AsPC-1 cells, so we chose siRNA-3 for the following studys (Fig. 2A, B). We silenced hsa_circ_0001846 and performed functional studies in two PC cell lines (PANC-1 and AsPC-1). First, scratch experiments showed that silencing hsa_circ_0001846 caused a significant weakeness in the migration ability of PC cells compared to the NC group (Fig. 2C, D). CCK-8 and EdU experiments also demonstrated that the proliferation of PC cells was inhibited after transfecting si-hsa_circ_0001846 (Fig. 2E–H). We also simulated the cell invasion process by transwell experiment with matrix glue to proved that silencing hsa_circ_0001846 reduced the number of cells passing through the chamber micropores and decreased the invasion ability of PANC-1 and AsPC-1 cells (Fig. 2I, J).

**Fig. 3:**
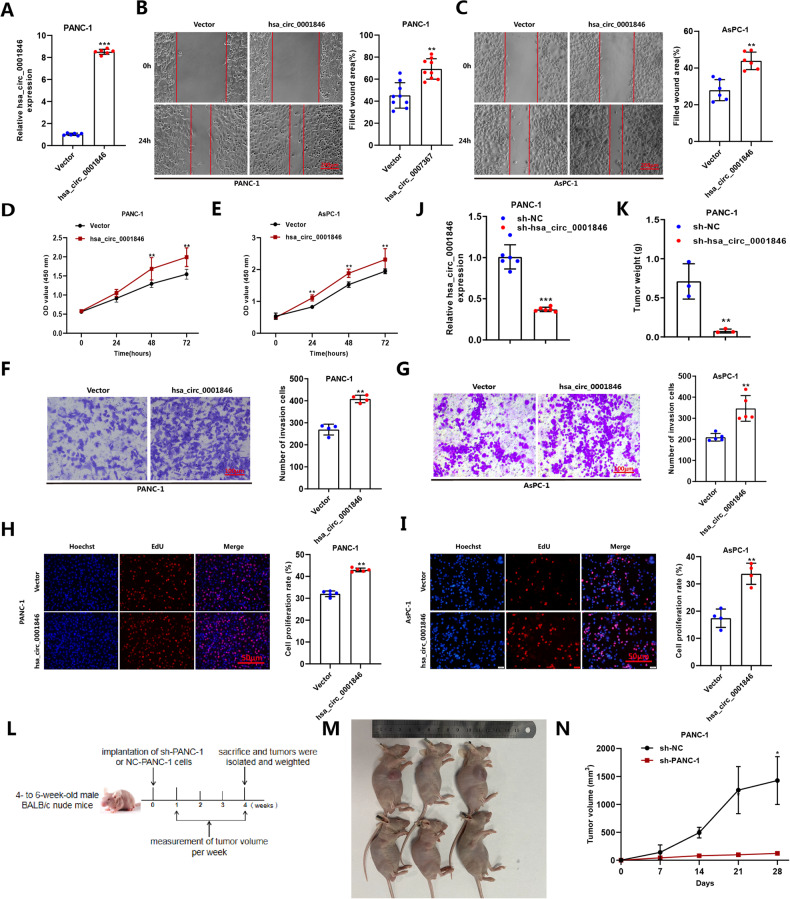
Hsa_circ_0001846 promotes invasion, proliferation and migration of PC cells in vivo and vitro. **A** Establishing overexpression plasmid of hsa_circ_0001846 and detecting the plasmid efficiency in PANC-1 by qRT-PCR. **B–I** Transfecting pCE-RB-Mam-EGFP-hsa_circ_0001846 and vector to generate wound-healing experiment (**B**, **C**), CCK-8 (**D**, **E**), Transwell (**F**, **G**) and EdU (**H**, **I**) in PANC-1 and AsPC-1. **J** Knocking out hsa_circ_0001846 in PANC-1 cells and amplifing by qRT-PCR. **K** Tumor weight of xenograft after knockdown of hsa_circ_0001846 compared with sh-NC. **L**, **M** Sh-NC and sh-hsa_circ_0001846 cells were subcutaneously injected into nude mice to establish the xenograft mode. **N** After successful modeling, the subcutaneous tumor volume was measured weekly. All data are shown as the mean ± SD of at least three independent experiments. *p < 0.05, **p < 0.01, ***p < 0.001.

On the contrary, to more strongly prove the role of hsa_circ_0001846 on the biological functions of PC cell lines, we constructed the overexpression vector pCE-RB-Mam-EGFP-hsa_circ_0001846 and verified the overexpression efficiency with qRT-PCR (Fig. 3A). The scratch assay showed that the overexpression of hsa_circ_000184 promoted the migration of PC cell lines (Fig. 3B, C). Meanwhile, CCK-8, EdU and Transwell experiments also demonstrated that overexpression of hsa_circ_0001846 promoted cell proliferation and invasion (Fig. 3D–I). Next, PANC-1 cell line with stable knockout of hsa_circ_0001846 was constructed in order to explore whether hsa_circ_0001846 affected the growth of PC cells in vivo (Fig. 3J). We resuspended sh-PANC-1 and sh-NC cells with matrix gel and injected them into the subcutaneous tissue of nude mice to construct nude mouse xenograft models (Fig. 3L). Four weeks later, nude mice were sacrificed, meanwhile we collected and measured the tumor tissues. We observed that the volume and weight of xenografts from sh-hsa_circ_0001846 cells were significantly smaller and lighter compared with sh-NC (Fig. 3K, M, N).

### Hsa_circ_0001846 is a sponge for miR-204-3p

**Fig. 4:**
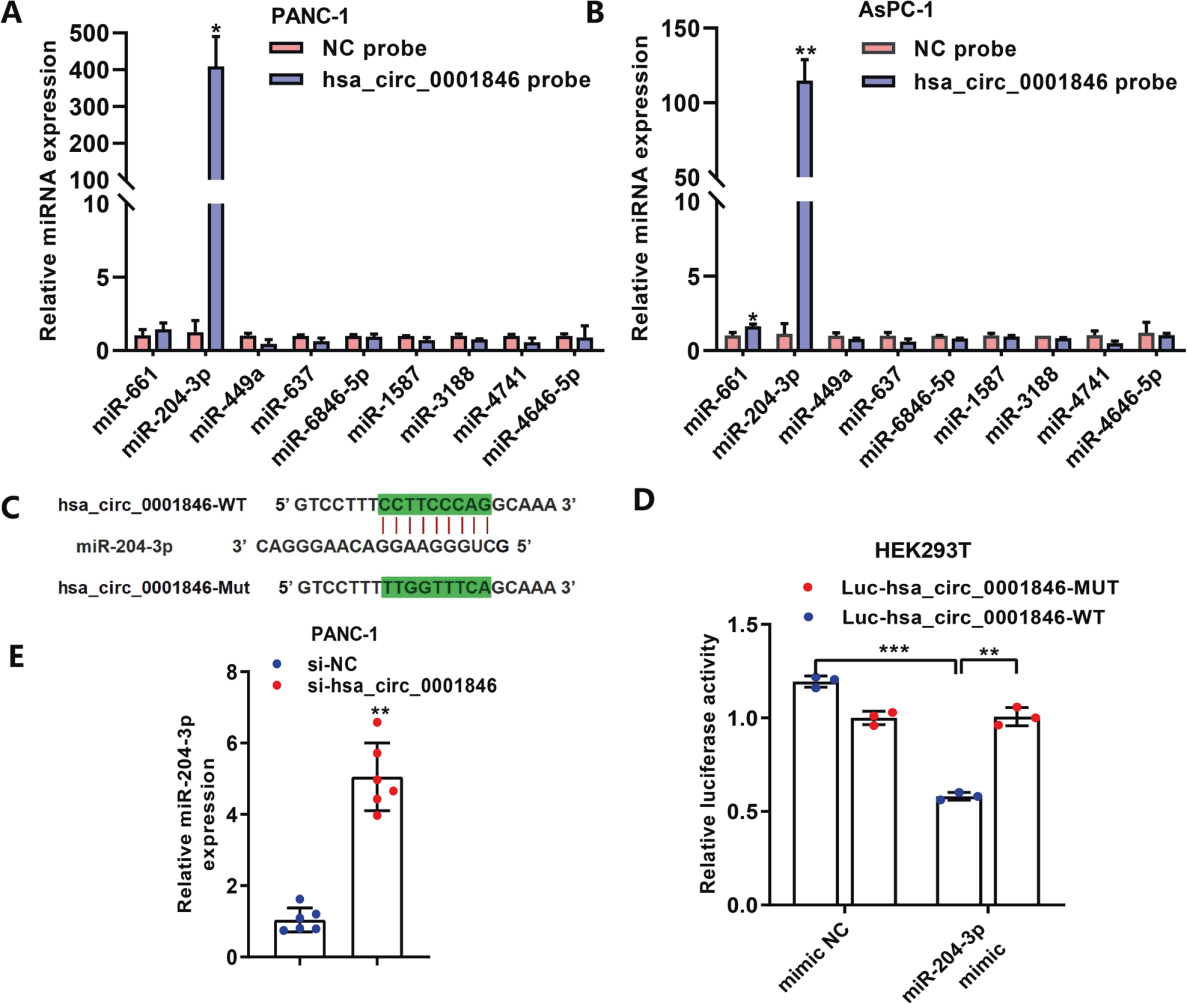
Hsa_circ_0001846 acts as a sponge for miR-204-3p. **A**, **B** Candidate microRNAs were pulled down and enriched with hsa_circ_0001846 probe and then detected by qRT-PCR. **C** The prediction for miR-204-3p binding sites on hsa_circ_0001846 transcript. **D** The luciferase activities of the hsa_circ_0001846 luciferase reporter vector (WT or Mut) in HEK293T cells transfected with miR-204-3p mimic or mimic NC. **E** The expression level of miR-204-3p in PANC-1 cells transfected with si-hsa_circ_0001846 by qRT-PCR. All data are shown as the mean ± SD of at least three independent experiments. *p < 0.05, **p < 0.01, ***p < 0.001.

Through the above in vivo and vitro functional experiments, we determined that hsa_circ_0001846 has a promoting effect on PC deterioration. To further clarify how hsa_circ_0001846 regulates PC progression, we performed mechanistic studies. Previous more studies have reported that circRNA mainly acts as a sponge of miRNAs to regulate the expression of the corresponding downstream target proteins. The miRNAs target prediction softwares, including Circinteractome, Starbase and Circbank, were used to predict the potential targeting miRNAs of hsa_circ_0001846. We also performed the RNA pulldown assay by designing a specific biotin probe for hsa_circ_0001846. The regulatory effect of hsa_circ_0001846 on the candidate miRNAs was explored. The results showed that miR-204-3p was significantly accessible to be catched by hsa_circ_0001846 than the other candidate miRNAs (Fig. 4A, B). Subsequently, we mutated the site of hsa_circ_0001846 bound to miR-204-3p to performed dual-luciferase reporter assay and found that miR-204-3p mimic significantly reduced the luciferase activity of WT-hsa_circ_0001846 vector, while the luciferase activity of Mut-hsa_circ_0001846 vector did not change (Fig. 4C, D). Meanwhile, qRT-PCR results indicated that silencing hsa_circ_0001846 upregulated the expression level of miR-204-3p in PANC-1 (Fig. 4E). The above experimental results proved that there are binding and regulatory relationships between hsa_circ_0001846 and miR-204-3p.

### The effect of miR-204-3p on the biological function of PC cells is the opposite of hsa_circ_0001846

**Fig. 5:**
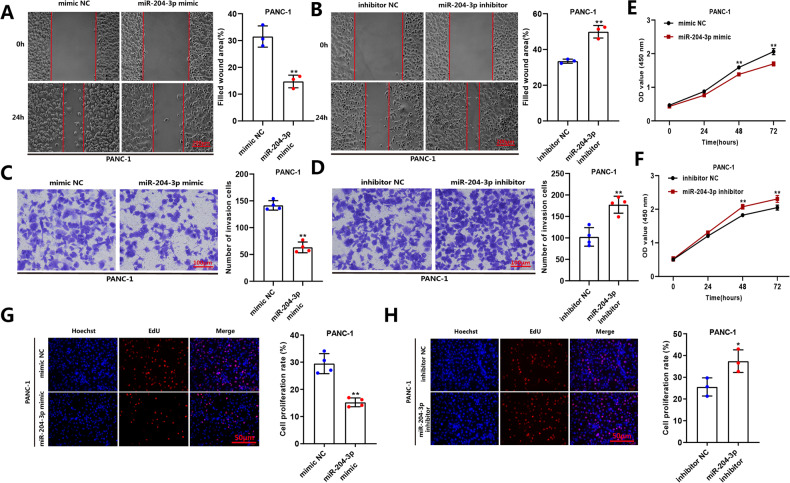
Functional experiments evaluate the abilities of miR-204-3p on proliferation, migration, and invasion of PC cells. **A**, **B** Cell migratory capabilities was determined by wound-healing in PANC-1 cells transfected with miR-204-3p inhibitor or mimic. **C**, **D** Cell invasive capabilities was determined by transwell invasion assays with matrigel in PANC-1 cells transfected with miR-204-3p inhibitor or mimic. **E**–**H** EdU and CCK-8 assay were used to evaluate the proliferation ability of the PANC-1 cells transfected with miR-204-3p inhibitor or mimic. All data are shown as the mean ± SD of at least three independent experiments. *p < 0.05, **p < 0.01, ***p < 0.001.

Since the role of miR-204-3p in PC has been rarely explored, its functions were further investigated in the PANC-1 and AsPC-1 cell lines. Wound-healing assay showed that transfection of miR-204-3p mimic in cells inhibited the migratory capacity of PC cells (Fig. 5A), while an inhibitor of miR-204-3p promoted cell migration (Fig. 5B). The same results were shown in other functional experiments, CCK-8 and EdU experiments showed that miR-204-3p mimic inhibited the proliferation capacity of cells, while miR-204-3p inhibitor promoted cell proliferation (Fig. 5C–F). Transwell experiment demonstrated that treatment of cells with miR-204-3p mimic inhibited cell invasion, while the miR-204-3p inhibitor promoted cell invasion (Fig. 5G, H). These results indicated that in contrast to hsa_circ_0001846, miR-204-3p inhibited the proliferation, migration and invasion of PC cells.

### MiR-204-3p inhibitor reverses the effects of si-hsa_circ_0001846 on proliferation, migration, and invasion in PC cells

**Fig. 6:**
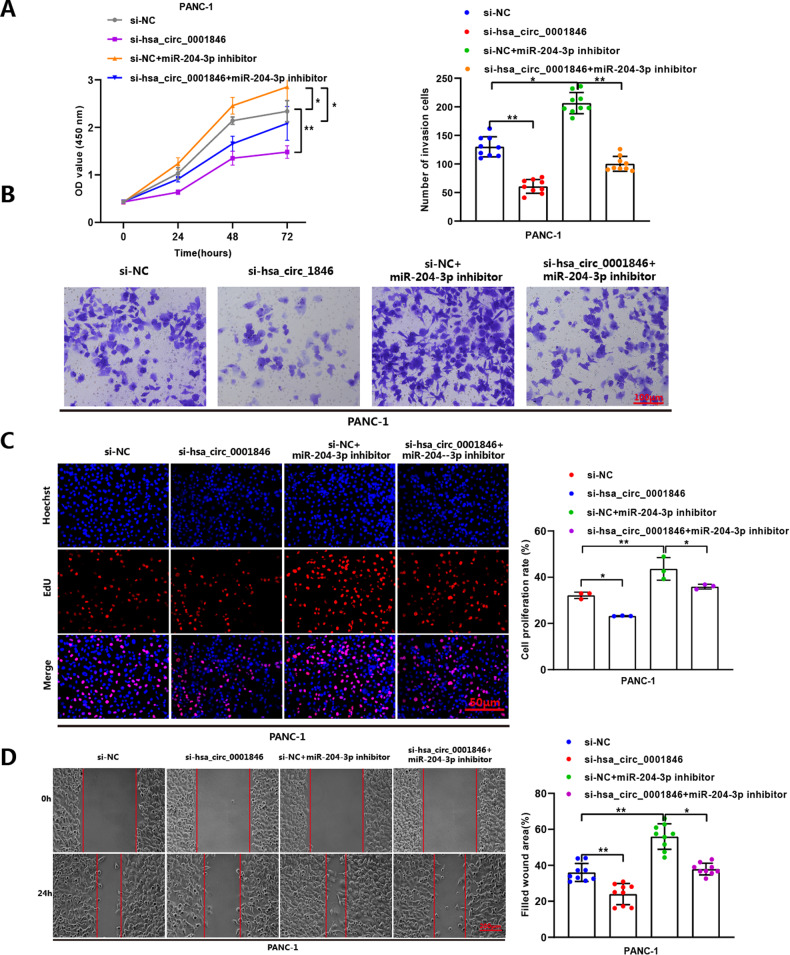
MiR-204-3p inhibitor reverses the effects of si-hsa_circ_0001846 on proliferation, migration, and invasion in PC cells. CCK-8 (**A**), Transwell invasion (**B**), EdU (**C**), and wound-healing (**D**) assays demonstrated that co-transfection with the miR-204-3p inhibitor could reverse the proliferation, migration, and invasion ability of PANC-1 cells after treated with si-hsa_circ_0001846. All data are shown as the mean ± SD of at least three independent experiments. *p < 0.05, **p < 0.01, ***p < 0.001.

To further demonstrate that hsa_circ_0001846 regulated PC progression by sponging miR-204-3p, we co-transfected si-hsa_circ_0001846 and miR-204-3p inhibitor in PANC-1 cells. Functional experiments showed that compared with the NC group, silencing hsa_circ_0001846 attenuated the migration, proliferation and invasion abilities of PANC-1 cells, while miR-204-3p inhibitor enhanced these abilities. At the same time, compared with si-hsa_circ_0001846 group, the migration, proliferation and invasion abilities of PANC-1 cells were saved by transfecting miR-204-3p inhibitor (Fig. 6A–D). The above results indicated that miR-204-3p is a key acting molecule downstream of hsa_circ_0001846.

### The hsa_circ_0001846 exerts its carcinogenesis effects by indirectly regulating the expression level of KRAS by miR-204-3p

**Fig. 7:**
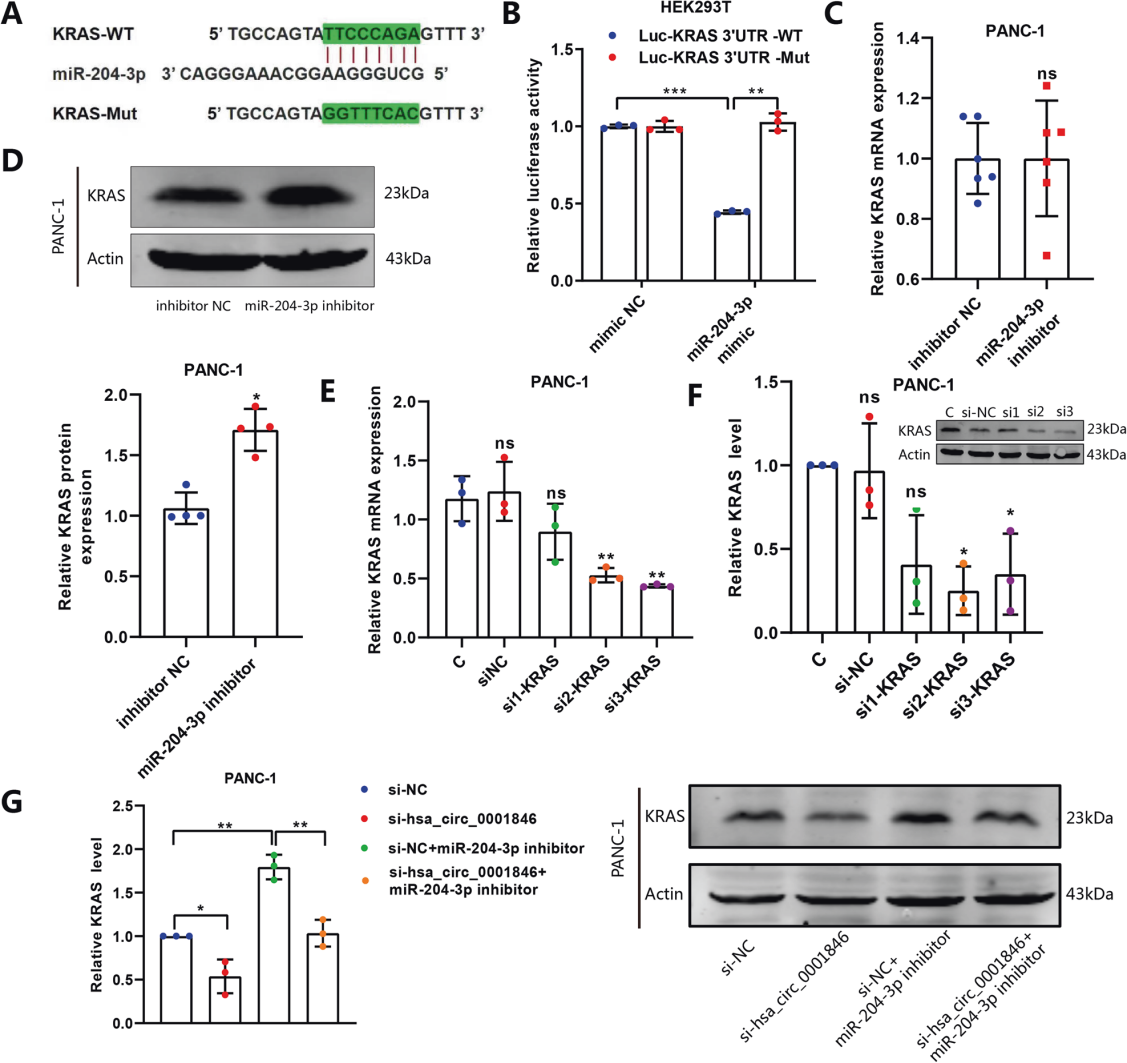
KRAS is a downstream target of miR-204-3p. **A** Schematic illustration of the wild-type (WT) and mutant (Mut) KRAS luciferase vectors. **B** The luciferase activities of the KRAS luciferase reporter vector (WT or Mut) in HEK293T cells transfected with miR-204-3p mimic or mimic NC. **C** The mRNA expression of KRAS was measured by qRT-PCR in PANC-1. **D** The protein level of KRAS was detected by western blot in PANC-1 transfected miR-204-3p inhibitor. **E**, **F** Designing and synthesizing the three small interference RNAs of KRAS and detecting the silencing efficiency in PANC-1 by qRT-PCR and western blot. **G** Expression level of KRAS was detected by western blot. All data are shown as the mean ± SD of at least three independent experiments. *p < 0.05, **p < 0.01, ***p < 0.001.

**Fig. 8:**
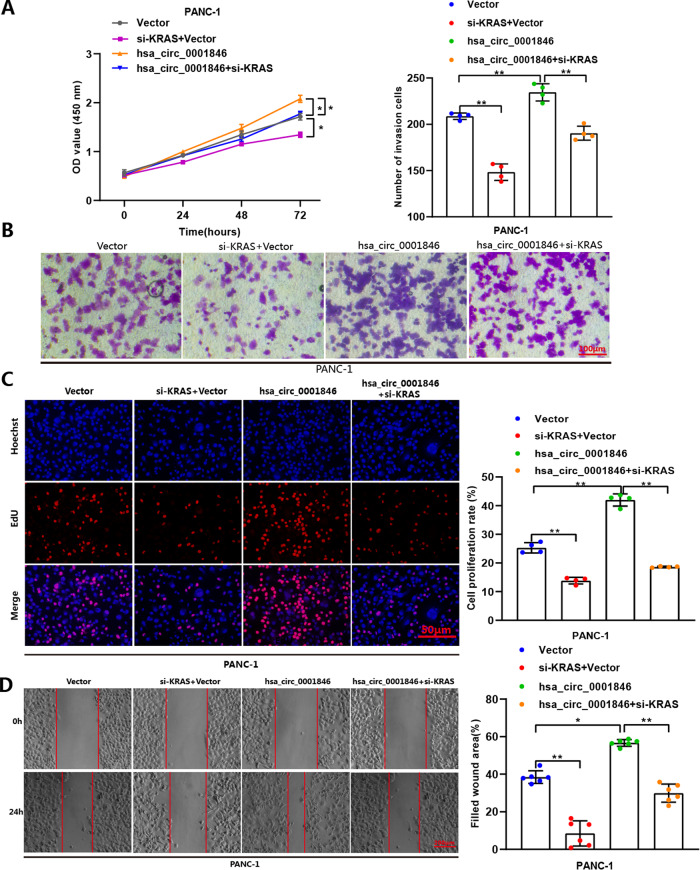
The effects of overexpression hsa_circ_0001846 could be reversed by co-transfection with si-KRAS. CCK-8 (**A**), transwell invasion (**B**), EdU (**C**), and wound-healing (**D**) assays were conducted by co-transfection with hsa_circ_0001846 and si-KRAS in PANC-1. All data are shown as the mean ± SD of at least three independent experiments. *p < 0.05, **p < 0.01.

A large number of previous studies have confirmed that KRAS is one of the oncogenes involved in PC progression. We used the bioinformatics websites of miRDB, miRDIP, miRWALK and TargetScan to analyze the sequence of miR-204-3p and KRAS and found a binding relationship between them (Fig. 7A). Therefore, we mutated the binding site of miR-204-3p and KRAS, constructed the Mut-KRAS/WT-KRAS plasmid and performed dual-luciferase experiment, which showed that HEK293T cells with co-transfected WT-KRAS and miR-204-3p mimic significantly reduced the luciferase activity, while Mut-KRAS and miR-204-3p mimic did not significantly change the luciferase activity in the cells (Fig. 7B, C). Although we found that miR-204-3p inhibitor did not significantly increase the mRNA level of KRAS, the western blot results showed that it was able to increase the protein expression level of KRAS, suggesting that miR-204-3p may primarily affect the post-transcriptional level of KRAS (Fig. 7D, E). Simultaneously, silencing hsa_circ_0001846 inhibited KRAS expression, and miR-204-3p inhibitor antagonized this effect (Fig. 7F–H). Functionality experiments also showed that silencing KRAS can reverse the cancer-promoting effect caused by overexpression of hsa_circ_0001846 (Fig. 8A–D).

The above results indicated that KRAS is a downstream target of miR-204-3p, and hsa_circ_0001846 indirectly regulated KRAS expression by sponging miR-204-3p, thus exerting its cancer-promoting effect.

## Discussion

Compared to other cancers, PC has a very poor prognosis, with only 24% of patients surviving one year after diagnosis and only 9% surviving five years. In early stages, PC usually has no symptoms [24]. As the tumor progresses, it gradually exhibits non-specific symptoms, including jaundice, light-colored stools, abdominal pain, weight loss and fatigue, which makes PC become one of the most misdiagnosed and missed diseases [25]. Up to now, there are several diagnostic and staging criteria tools available, such as pancreatic CT [26, 27], MRI, endoscopic ultrasound guided fine needle aspirin as well as cytological diagnostic techniques with sensitivity up to 80% [24, 28]. In symptomatic patients, blood levels of cancer antigen 19-9 assisted diagnosis and predicted postoperative prognosis and recurrence [29], but it was not PC-specific and don’t be used as a biomarker for PC prevention and early screening [30]. Therefore, in the face of this situation, this study aims to find biomarkers that can be used as an individual screening tool for asymptomatic patients, so as to achieve early prevention and treatment of PC and improve the survival rate.

Numerous studies have confirmed that circRNAs play an important role in a variety of cancers. CircRNAs are generated by reverse shearing of their original genes, so they are functionally similar to the original genes. Previous studies have shown that UBAP2 contains a ubiquitin-associated (UBA) domain and damages the structure and function of target proteins [31, 32]. UBAP2 was highly expressed in PC and involved in regulating the cancer progression [33–35]. Hsa_circ_0001846 originated from the UBAP2, so we put forward whether it can also affects the development of the PC. Subsequently, we found that hsa_circ_0001846 was highly expressed in cell lines and tissues of PC, and functional experiments in vivo and vitro indicated that hsa_circ_0001846 promoted the proliferation, invasion and migration of PC.

In 2013, ciRS-7 was first reported to directly bind and adsorb miR-7 in a sequence-specific manner and indirectly up-regulate the expression of downstream target genes of miR-7 [36]. Since then, competitive endogenous mechanism has become the most widely studied involvement mechanism of circRNAs. Previous studies also indicated that circRNAs play a role through different mechanisms depending on their location in cells. Therefore, we found that hsa_circ_0001846 was located in cytoplasm by FISH experiment. This suggested that it was involved in sponge mechanisms or directly binding proteins to regulate downstream oncogene targets. The use of multiple databases about cancer and circRNAs has made the process of predicting downstream targeted genes for circRNAs more efficient, faster, and scientific. We predicted the potential miRNAs that cound bind to hsa_circ_0001846 through the multiple databases including Starbase, Circbank and CircRNA Interactome. After comprehensive analysis, it was concluded that hsa_circ_0001846 may interact with 9 miRNAs. The potential binding compounds were screened and verified by RNA pulldown and Dual-luciferase experiments. The results showed that miR-204-3p was significantly enriched by hsa_circ_0001846 in PANC-1 and AsPC-1 cells. In view that the effects of miR-204-3p on PC have rarely been independently studied, we constructed its mimic and inhibitor for functional gain and loss experiments. The results demonstrated that, in contrast to hsa_circ_0001846, miR-204-3p had inhibitory effects on the malignant behavior of PC cells. Moreover, inhibition of miR-204-3p canceled the antitumor effect caused by silencing hsa_circ_0001846. This proved that miR-204-3p was the main target for hsa_circ_0001846 to play a role in PC.

It is well known that the activating mutations of small GTPases, such as KRAS, near universal in PC and critical for pathogenesis [37]. UBAP2 has been reported to inhibit the activation of KRAS and thus the growth of PC [35]. As UBAP2 is the original gene of hsa_circ_0001846, we speculated that it may also have a regulatory effect on KRAS. Database target prediction and double fluorescin assay confirmed the binding relationship between KRAS and miR-204-3p, further qRT-PCR and western blot experiments indicated that miR-204-3p only affected the post-transcriptional level of KRAS, but had no regulatory effect on its mRNA. At the same time, we found that miR-204-3p inhibitor cancel the inhibition effect of silencing hsa_circ_0001846 on KRAS, and knockout of KRAS reversed the cancer-promoting effect caused by overexpression of hsa_circ_0001846. Therefore, our results showed that hsa_circ_0001846 binded to miR-204-3p, releasing the inhibition of miR-204-3p on KRAS and promoting the progression of PC. CircRNAs are considered to have the potential of clinical biomarkers because they are not only stable in the blood, but also function in body fluids and exosomes after being released by cells [38], facilitating clinical sampling and detection. Therefore, our investigation confirmed the potential of hsa_circ_0001846 as a PC-specific clinical biomarker, providing more options and theoretical basis for exploring the diagnosis and treatment targets of PC.

## Materials and methods

### Clinical tissure

25 pairs of fresh frozen PC and adjacent nontumorous tissues were acquired from the Harbin Medical University Cancer Hospital (Harbin, China). All the patients had not undergone chemotherapy, radiotherapy, or immunotherapy before surgery. All clinicopathological diagnoses were confirmed by two pathologists. This study was approved by the Ethics Committee of Harbin Medical University (IRB3002720).

### RNA extraction, reverse transcription, and quantitative real-time PCR analysis (qRT-PCR)

The cells were cultured in 12-well plates and the tissue was frozen with liquid nitrogen and then ground with a Motor-Driven Tissue Grinder Using Trizol reagent (Invitrogen, California USA) extract Total RNA from PC cell lines and tissues. Then the extracted RNA was reverse-transcribed on the T100 Thermal Cycler (BIO-RAD, California USA) using Prime Script RT reagent Kit (Toyobo, Osaka Japan) to obtain single-stranded cDNA. Finally, refer to the manufacturer’s instructions, quantitative PCR analysis was performed using the SYBR Green Mix kit (TaKaRa, Dalian, China) to obtain the expression levels of relevant RNAs. The process was performed on an ABI 7500 Real-Time PCR system (Applied Biosystems, California USA), and β-actin were used as internal parameters, the relative folding changes of miRNAs, circRNAs and mRNAs were calculated by 2^−△△CT^.

### Western blot

Cultivating PANC-1 or AsPC-1 cells in the six-well plates and extracting total protein with RIPA lysis buffer (Beyotime, Shanghai, China). After ultrasonic treatment with cell lysates for three times (5 min each time), the cell lysates were treated with BCA Protein Analysis Kit (Beyotime, China), and protein concentration was detected by enzyme-labeled instrument (Molecular Devices, California, USA). Proteins were used to SDS-page and transferred to nitrocellulose filter membrane (Pall Corporation, New York, USA), blocked with milk for 1 h and incubated overnight with primary antibody at 4 °C. On the second day, PBST was used to clean it for three times, each time for 10 min, and then incubated with secondary antibody for 40 min. Finally, Odyssey infrared imaging instrument (LI-COR, New Jersey, USA) was used to scan and analyze the membrane. Antibodies used in this study include: anti-β-actin (1:20000, ABclonal, Wuhan, China), anti-KRAS (1:1500, Cell Signaling Technology, Danvers, USA), secondary antibody (800 R rabbit antibody, 1:10 000, LI-COR, New Jersey, USA).

### Cell culture and transfection

PANC-1 and BxPC-3 cells were cultured in DMEM medium, AsPC-1 and HPDE cells were cultured in RPMI-1640 medium, Capan-1 cells were cultured in IMDM medium, and SW-1990 cells were cultured in L-15 medium. Every kind of medium was supplemented with 1% Penicillin-Streptomycin-Amphotericin B Solution and 10% fetal bovine serum. The cells of PANC-1, AsPC-1, BxPC-3 and Capan-1 were cultured in an incubator at 37 °C and 5% carbon dioxide, while the cell of SW-1990 was cultured in an incubator at 37 °C and 100% air. The human PC cell lines AsPC-1, BxPC-3, Capan-1, PANC-1, SW-1990 cells were purchased from Chinese Academy of Sciences, and human pancreatic ductal epithelial (HPDE) cells were purchased from Zeye Biotechnology. The cells authenticated by short tandem repeat (STR) profiling, and tested free from mycoplasma. For transfection under the condition of avoiding light, the plasmids and transfection reagents were diluted respectively by Opti-MEM. After 5 min, the two were mixed and incubated together for 15 min, then added to the culture plate. After 6-8 h, the transfection reagents and empty culture were discarded and cells were cultured in complete culture medium. According to different research projects, cells were treated at different time nodes for follow-up experiments. MiR-204-3p mimic or inhibitor and their corresponding controls were purchased from General Biosystem Company (Anhui, China). Si-hsa_circ_0001846, si-KRAS and the forced expression vector of hsa_circ_0001846 were designed synthesized by RiboBio Biotechnology (Guangzhou, China). Transient transfections were conducted using Lipofectamine 2000 (Invitrogen, California, USA) and X-treme (Roche, Basel, Switzerland).

### Xenograft tumor model

Stable knockdown hsa_circ_0001846 PANC-1 cells were constructed by Hanbio Biotechnology (Shanghai, China). Ten four-week-old male BCLB/c mice (Charles River, Beijing, China) were randomly and equally divided into two groups. 5 × 10^7^ sh-hsa_circ_0001846 or sh-NC cells were digested and resuspended with 200 μl of highly concentrated stromal gel and injected subcutaneously in the axilla of mice. Tumor volumes (length × width^2^/2) were measured weekly, and mice were executed after four weeks, and tumors were separated for weighing and photography. This study was approved by the Ethics Committee of Harbin Medical University.

### Cell counting Kit-8 (CCK-8) assay

According to the manufacturer’s protocol, using Cell Counting Kit-8 Kit (CCK-8) assay (Meilunbio, Dalian, China) assess the viability of PC cells (PANC-1 and AsPC-1). Transfection was performed when the cell density was 60-70%. After being transfected, cells were cultured in 96-well culture plates for 0, 24, 48, and 72 h. Next cells were incubated at 37 °C for 2 h with 200 µl cck-8 reagent each well, and then absorbance was measured at 450 nm using a microplate reader (Molecular Devices, California, USA).

### 5-ethyl-2’-deoxyuridine (EdU) assay

According to the manufacturer’s instructions, an EdU assay kit (Ribobio, Guangzhou, China) was used to detect the proliferative function of PANC-1 and AsPC-1 cell lines. The cells were cultured in 24-well plates, incubated with 300 µl EdU (50 µM) for 2 h, and fixed with 4% paraformaldehyde. The newly proliferating cells and nuclear were then labeled with Apollo and Hoechst33342 staining, respectively. The slices were removed from a 24-well plate, sealed with an anti-fluorescence quencher and stored in a wet box at 4 °C. It is then observed and photographed under a fluorescent microscope (Zeiss Axio Scope A1, Baden-Wurttemberg, Germany).

### Wound-healing assay

PANC-1 or AsPC-1 cells were inoculated in six-well plates. Then transfection was performed when the cell growth reached 70%. The wound was made with a 10 μl-pipette tip, and photographs were taken by the microscope (Olympus, Tokyo Japan) at ×100 magnification at 0 h and 24 h, respectively.

### Transwell invasion assays

The transfected cells were digested and resuspended in serum-free medium, and 200 μl cell suspension (containing 5 × 10^5^ cells) was added to the upper chamber of the matrix gel (Corning, New York, USA) coated chamber (aperture = 8 nm) in a 24-well plate. In order to promote cell invasion, 500 μl serum-containing medium was added into the lower compartment and then to expel the air bubbles between the upper and lower compartments. 24 h later, the cells passing through the surface of the lower chamber were fixed with 700 μl methanol pre-cooled in the refrigerator at 4 °C for 20 min, the upper chamber was cleaned with PBS, the upper chamber was wiped with cotton swabs, the upper chamber was dried upside down, and then stained with 500 μl crystal purple for 15 min. After drying, the bottom membrane of the chamber was cut off with a knife, sealed with gum and photographed under ×100 field of view under Zeiss AxioScopeA1 microscope (Carl Zeiss AG, Baden-Wurttemberg, Germany) to calculate the number of invading cells.

### RNA pulldown assay

Hsa_circ_0001846 RNA probe was designed and synthesized by Ribobio (Guangzhou, China). The beads were first pretreated and incubated with the hsa_circ_0001846 probe to prepare streptavidin-labeled beads. PANC-1 cell lysates were collected, and the beads were added for circRNA-RNA binding. Then RNase R was added for RNA purification followed by reverse transcription, and the abundance of relevant mircoRNA was analyzed by qRT-PCR.

### Dual-luciferase reporter assay

Targeted the complementary sequence in 3′-UTR between miR-204-3p and hsa_circ_0001846 or KRAS, and constructed the Mut/WT-psiCHECK-2 plasmid (Promega, Wisconsin, USA). Co-transfected the mimic or NC of miR-204-3p and Mut/WT-psiCHECK-2 plasmid of hsa_circ_0001846 or KRAS in HEK293T cells. After 48 h, the activities of firefly and Renilla luciferase in the cells were detected by luciferase reporter assay system (Promega, Wisconsin, USA).

### RNA fluorescence in situ hybridization (FISH)

Appropriate amount of cells were inoculated on the 24-well plate cell slide, and cell fixation and permeability were carried out when the cell growth density reached about 50%. Prepare the prehybrid solution, hybrid solution, 1× SSC, 2× SSC, and 4× SSC + 0.1% Tween-20 according to the kit instructions (RiboBio, Guangzhou, China). Each well was sealed with 200 μl prehybridization solution at 37 °C for 30 min. The hybrid solution was preheated at 37 °C. In the hybridization solution, 2.5 μl hsa_circ_0001846 probe (20 μM) (RiboBio, Guangzhou, China) was added away from light. After discarding the prehybridization solution in each well, 200 μl of hybridization solution containing probe was added, and the hybridization solution was kept away from light overnight at 37 °C. After cleaning the cells at room temperature and away from light. DNA was stained by 200 μl DAPI staining solution for 10 min. The cell slides were encapsulated and observed with confocal microscope.

### Agarose gel electrophoresis (AGE)

Configure the amplification system (Forward Primer: 0.5 μl, Reverse Primer: 0.5 μl, Transgene 2× Easy Taq Super Mix: 12.5 μl, Sterilized ddH_2_O: 8.5 μl, gDNA/cDNA: 300 ng), use the T100 Themal Cycle PCR instrument according to the set procedure. Use an analytical balance to weigh 0.5 g AGAR powder, heat and dissolve it with 50 ml 0.5× TAE solution, cool to room temperature, add 2 μl EB into the liquid level, stir well, pour into the rubber plate, insert the comb, gel for 30 min, and remove the comb. Pour the electrophoretic solution into the electrophoresis tank, add glue and 5 μl 6× DNA loading buffer into 10 μl amplified sample, and then load the sample in the well, with 7 μl DNA maker on each side of the well. The voltage is adjusted to 70 V, about half an hour later, the electrophoresis is finished, the gel is removed and scanned by machine (Tanon1600, Shanghai, China).

### Statistical analysis

The statistical analyses were mainly conducted using SPSS 16.0 (IBM, SPSS, Chicago, IL, USA) and GraphPad Prism 8.0 (GraphPad Software Inc., CA, USA). All data are presented as means ± standard deviation (SD). Researchers were blinded to the group allocation both during the experiment and/or when assessing the outcome. All experiments were repeated at least three times independently, and two-tailed unpaired *t* test was applied for two groups comparison analysis. *P* < 0.05 was considered statistically signifificant.

### Supplementary information

Original western blots

## Data Availability

The data generated or analyzed in the current study are available from the corresponding author upon reasonable request.
