# Supplementary material for: Circular RNA hsa_circ_0001846 facilitates the malignant behaviors of pancreatic cancer by sponging miR-204-3p and upregulating KRAS expression
Source: Cell Death Discov. 2023 Dec 11;9:448. doi: 10.1038/s41420-023-01733-2 (PMC10713563; doi:10.1038/s41420-023-01733-2)

Figure7D: n = 4

miR-204-3p

inhibitor

Inhibitor

NC

Inhibitor

NC

miR-204-3p

inhibitor

1. 2.


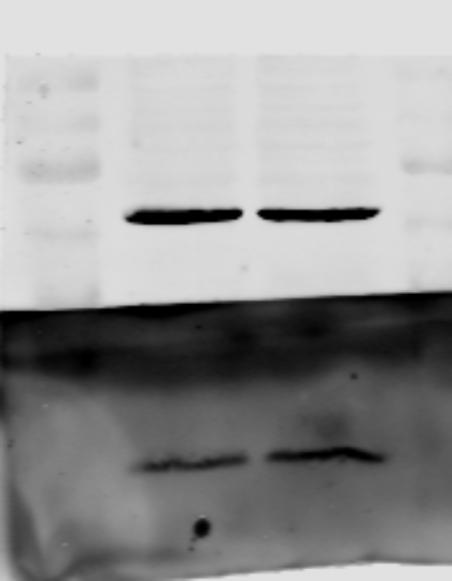


**55kDa**

**43kDa**

**34kDa**

**26kDa**

**16kDa**


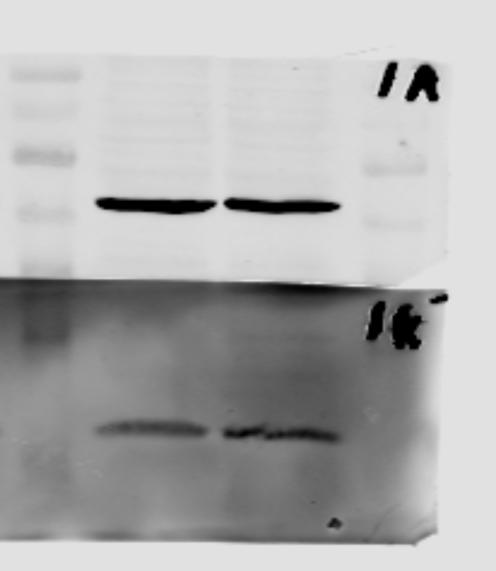


**55kDa**

**43kDa**

**34kDa**

**26kDa**

**16kDa**

Actin

Actin

KRAS

KRAS

Inhibitor

NC

miR-204-3p

inhibitor

miR-204-3p

inhibitor

Inhibitor

NC

3. 4.


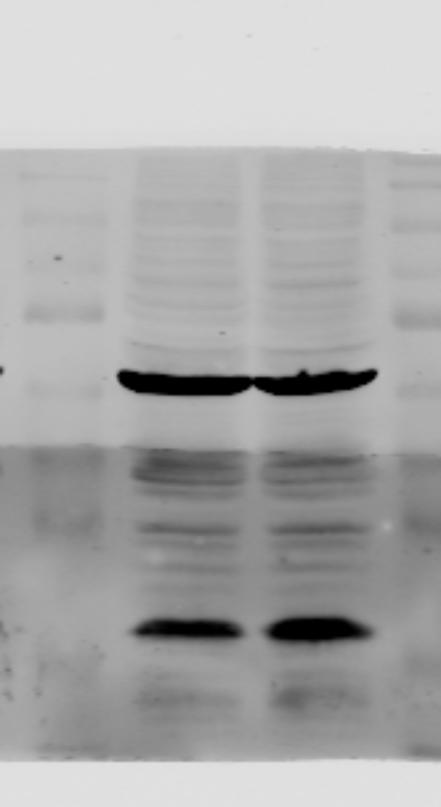


**55kDa**

**43kDa**

**34kDa**

**26kDa**

**16kDa**


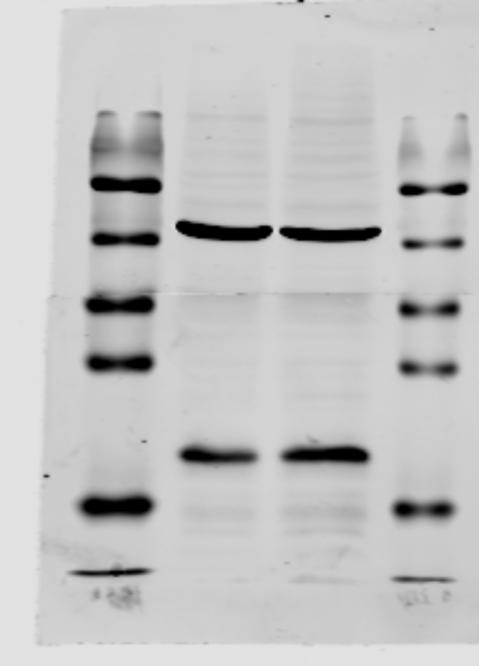


**55kDa**

**43kDa**

**34kDa**

**26kDa**

**16kDa**

Actin

KRAS

Actin

KRAS

1


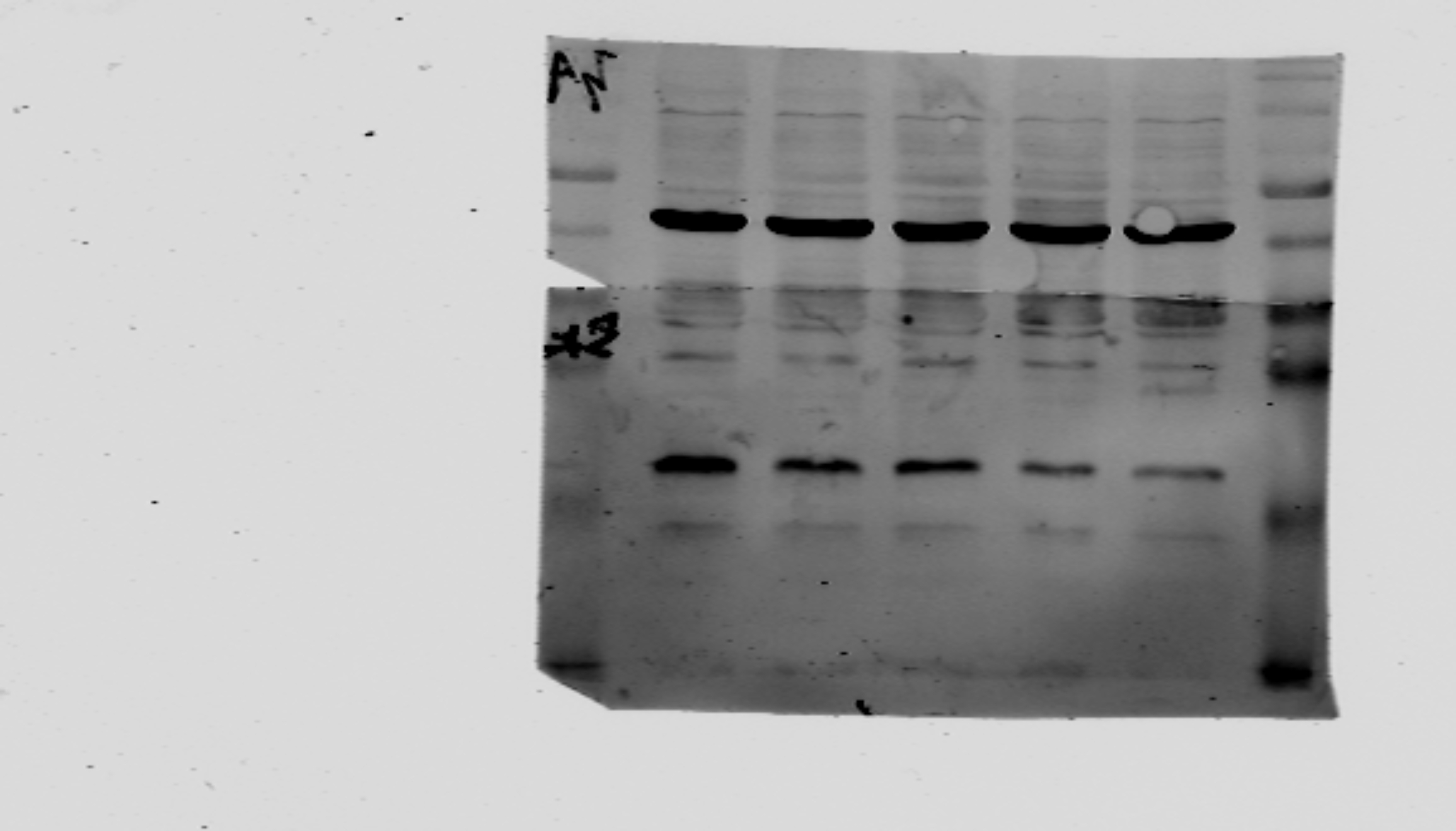


**55kDa**

**43kDa**

**34kDa**

**26kDa**

**16kDa**

C si-NC si1 si2 si3

Figure7E: n = 3

si-3

si-2

si-1

si-NC

C

si-3

si-2

si-1

si-NC

C

**55kDa**

**43kDa**

**34kDa**

**26kDa**

**16kDa**


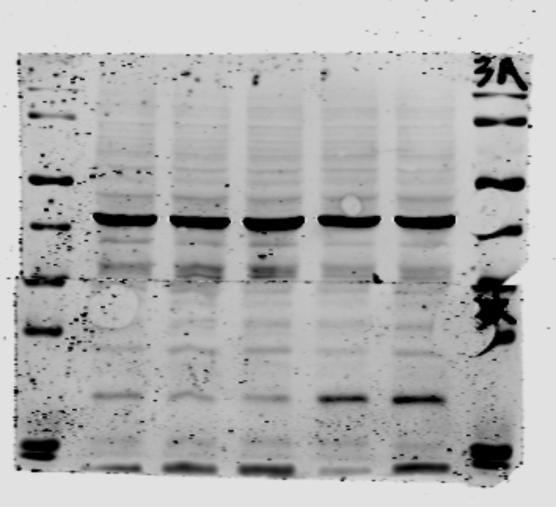

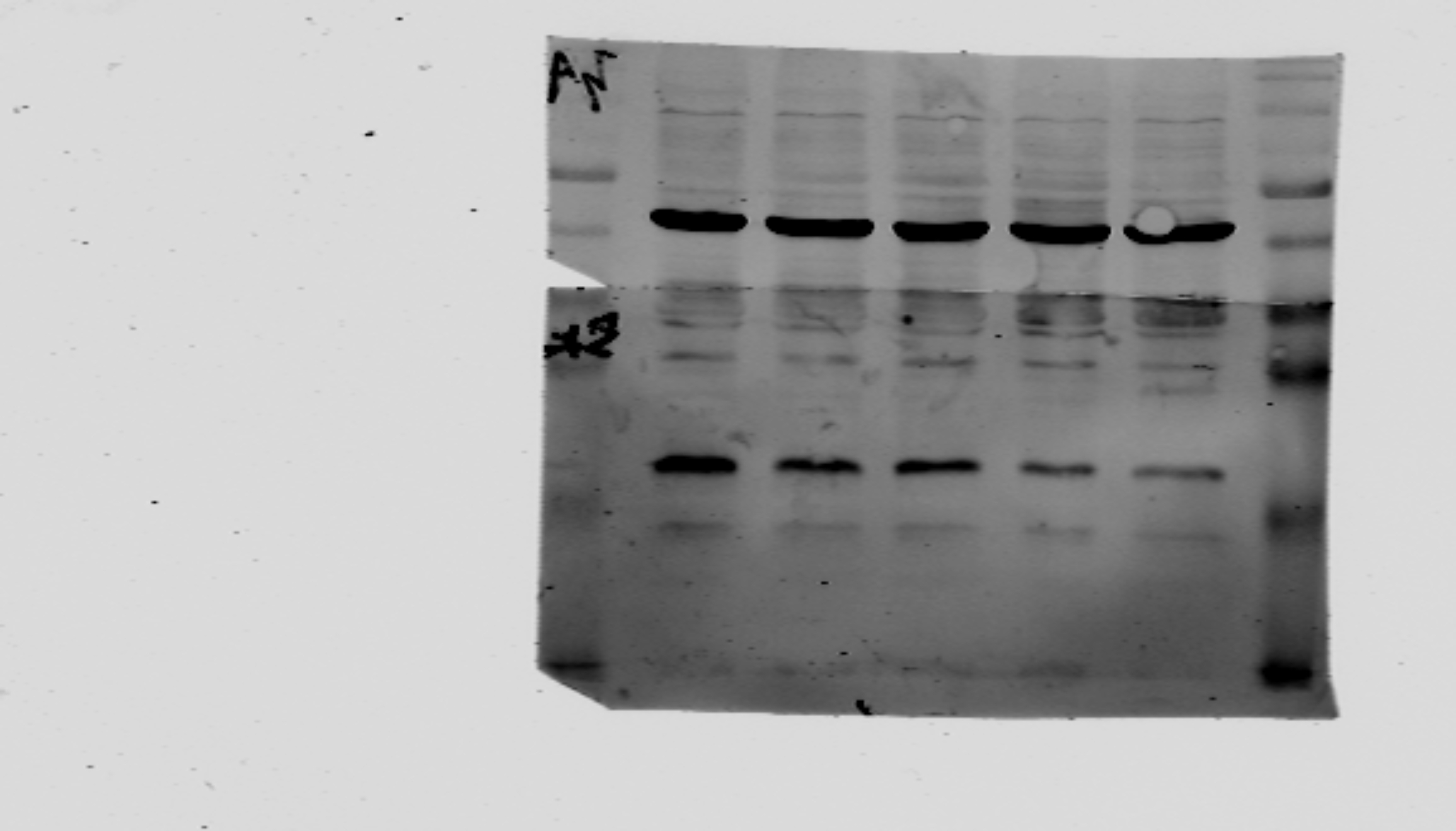


**55kDa**

**43kDa**

**34kDa**

**26kDa**

**16kDa**

Actin

Actin

KRAS

KRAS

si-NC

si-1

si-3

si-2

KRAS

Actin


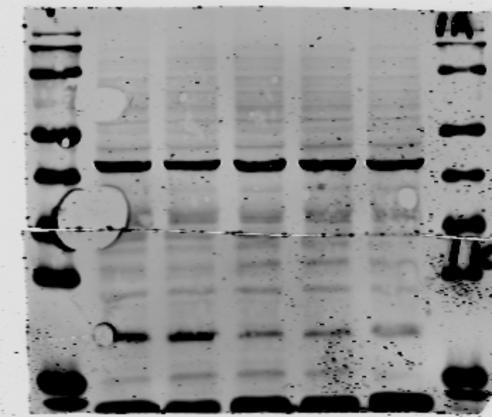


**55kDa**

**43kDa**

**34kDa**

**26kDa**

**16kDa**

si-hsa_circ_0001846+

miR-204-3p inhibitor

miR-204-3p inhibitor+

Figure7G: n = 3

miR-204-3p inhibitor

si-hsa_circ_0001846+

miR-204-3p inhibitor

miR-204-3p inhibitor+

miR-204-3p inhibitor

si-hsa_circ_0001846

si-hsa_circ_0001846

si-NC

si-NC

1. 2.

**55kDa**

**43kDa**

**34kDa**

**26kDa**

**16kDa**


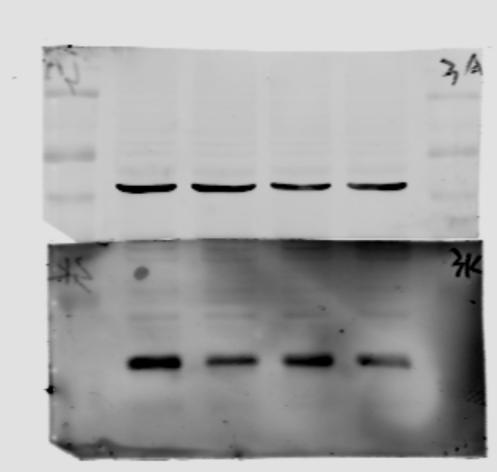


**55kDa**

**43kDa**

**34kDa**

**26kDa**

**16kDa**


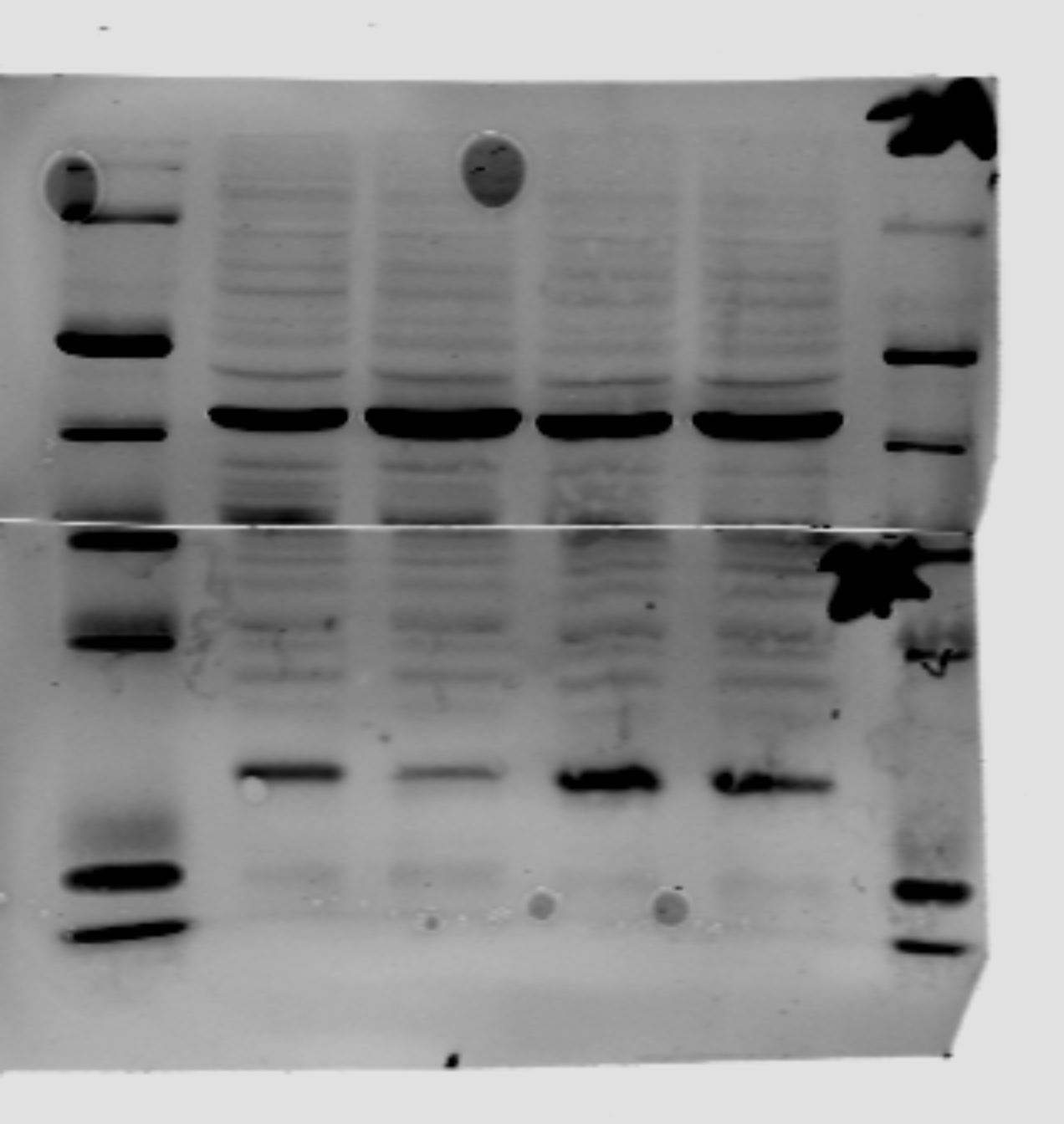


Actin

Actin

KRAS

KRAS

3.

Actin

KRAS

si-hsa_circ_0001846+

miR-204-3p inhibitor

miR-204-3p inhibitor+

miR-204-3p inhibitor

si-hsa_circ_0001846

si-NC

**55kDa**

**43kDa**

**34kDa**

**26kDa**

**16kDa**


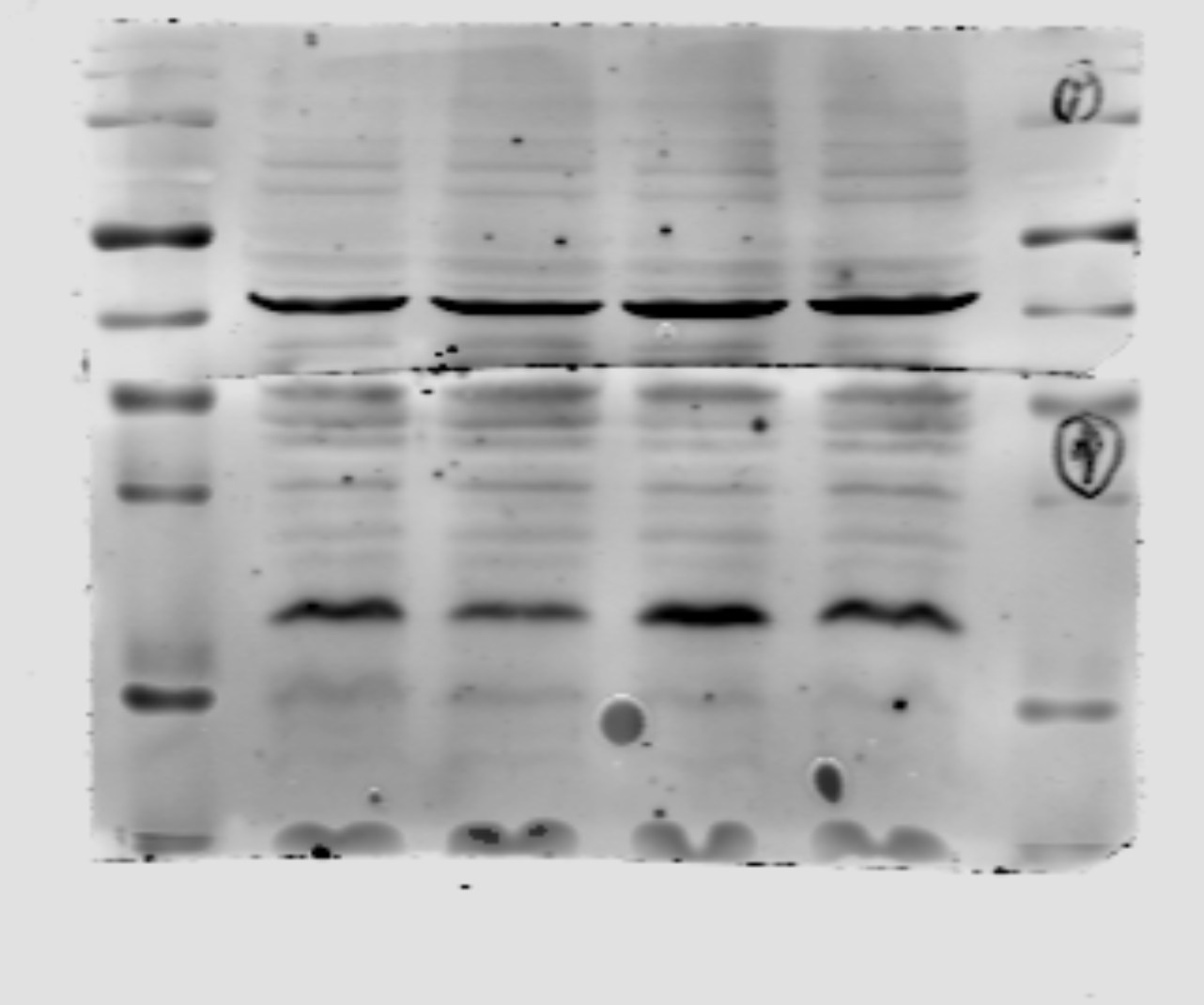

Supplement: Supplementary file 1 — Original western blots [file 41420_2023_1733_MOESM1_ESM.docx]
